# Low Power Systolic Array Based Digital Filter for DSP Applications

**DOI:** 10.1155/2015/592537

**Published:** 2015-04-02

**Authors:** S. Karthick, S. Valarmathy, E. Prabhu

**Affiliations:** ^1^Department of Electronics and Communication Engineering, Bannari Amman Institute of Technology, Sathyamangalam 638 401, India; ^2^Department of Electronics and Communication Engineering, Amrita Vishwa Vidyapeetham VLSI Design, Testing and Security Group, Coimbatore 641 112, India

## Abstract

Main concepts in DSP include filtering, averaging, modulating, and correlating the signals in digital form to estimate characteristic parameter of a signal into a desirable form. This paper presents a brief concept of low power datapath impact for Digital Signal Processing (DSP) based biomedical application. Systolic array based digital filter used in signal processing of electrocardiogram analysis is presented with datapath architectural innovations in low power consumption perspective. Implementation was done with ASIC design methodology using TSMC 65 nm technological library node. The proposed systolic array filter has reduced leakage power up to 8.5% than the existing filter architectures.

## 1. Introduction

DSP includes filtering, averaging, modulating, and correlating the signals in digital form to estimate characteristic parameter of a signal into a desirable form. DSP also does cardiac investigations through electrocardiogram (ECG) and extracts some characteristic parameters like QRS complexes for HRV (Heart Rate Variability) and *RR*-intervals (interval between two successive *R* complexes) [1].

Advancement in DSP has permitted many applications with unprecedented growth capabilities. Complex sensor and monitoring systems in biomedical applications implemented with general-purpose computing are highly sensitized to power consumption due to scaling of technologies, even preserving compute-intensive functions in dedicated hardware, due to scaled technology; the designers are forced towards power constrained designs. However, the power aware architectures are the solutions for the development of low power biomedical monitoring systems.

An example for low power biomedical application is digital hearing aid, a therapeutic device which must consume less power with very small system size fitting within the canals and should provide enough flexibility to implement dynamic range compression, directional processing, and other similar algorithms. Features like low power consumption, miniature size, and processing capability make the design suitable for many signal processing applications.

Even though the VLSI technology has rapid exponential growth characterized by Moore's law, still there is a boon in low power design parallel to technology scaling. This is due to use of existing circuit libraries and standard library cells which have inadequate circuit architectures for lower voltage and current operations. Inadequate design oriented/specific circuit architectures and lack of power aware cultures among the designers (due to lack of communication between the algorithm designers and low power chip designers) are also cause for the barriers of low power design [2].

Thus there is a need for power aware architectures to mitigate the low power constraint to certain extent. In this paper, the overview of the design has been evolved from the realization that many signal processing applications use filtering as the underlying component. Some of the algorithms that cast filtering framework are dynamic range compression, directional processing, and many others. Hence a systolic array based digital filter with datapath architectural innovation is implemented as the low power component in the DSP core for biomedical applications.

Several methods were adopted by the researchers in the past to reduce the power consumption. Distributed arithmetic based multiplier-less architecture was introduced to reduce the power consumption of the filtering component [3–5]. Other than the distributed arithmetic, the design specific systolic array architectures were also utilized to build the filter architecture. In [1], author has demonstrated the systolic array based digital filter for QRS detector of ECG analysis and has come up with common processing element. In [6] author has demonstrated a high speed 4 : 2 compressor architecture for any digital arithmetic integrated circuits. This digital filter is reimplemented in this paper with newer datapath architecture to achieve low power consumption. The designs were implemented with ASIC design methodology by mapping to 65 nm technological node using Synopsys Design compiler.

The remainder of the paper is organized as follows.  Section 2 describes the systolic array and characterizes different datapath circuit architectures utilized in the systolic array filter. Results are discussed and evaluated in Section 3.  Section 4 concludes the paper with the impact of datapath circuit behavior on the filter and references are provided in the last section.

## 2. Systolic Array and Its Datapath

The preprocessing stage of the QRS detection algorithm involves several digital filters. QRS detectors in the preprocessing stage have band-pass filters to reduce the noise, wave interference, and baseline wanders. The desirable pass-band for maximum QRS energy is approximately between 5 and 15 Hz. Center frequency of the pass-band filter is at 10 Hz and its amplitude response decides the spectrum of the average QRS complex to optimally pass the characteristic frequencies by attenuating the lower and higher frequencies. This is achieved by the cascade of low and high pass filter and their difference equations are given in the following:

(1) ynT=2ynT−T−ynt−2T+xnT −2xnT−6T+xnT−12T.

Difference equation can also be represented as follows:

(2) ynT=xnT−16T−132 ·ynT−T+xnT−xnT−32T.

Again the filters also can be represented as follows for computing one-dimensional recursive convolution:

(3) $$\begin{array}{c} H\left(z^{-1}\right)=\frac{\sum_{i=0}^{N}a_{i}z^{-i}}{1-\sum_{i=1}^{N}b_{i}z^{-i}}, \end{array}$$

where *a*_*i*_ (for *i* = 0 to *N*) and *b*_*i*_ (for *i* = 1 to *N*) are real coefficients. Thus the filters can be implemented by connecting array structures (consisting of adders, multipliers, and delay elements) continuously [1].

**Figure 1:**
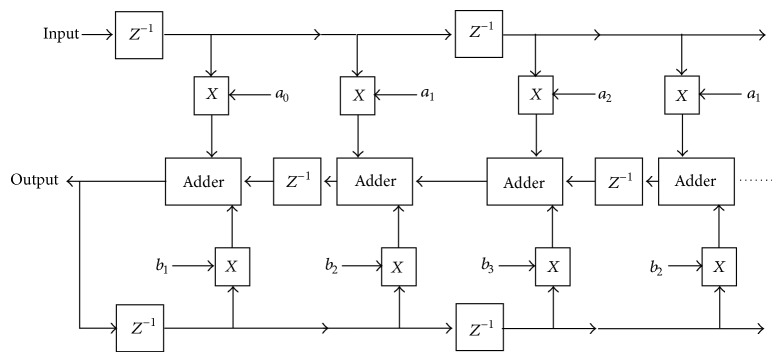
Systolic array architecture for digital filter.

Figure 1 shows that the systolic array architecture of digital filter contains adders, multipliers, and delay elements, where multiplier decides the overall performance of the array. As multiplier is the critical component and more in numbers, its efficiency will improve the overall efficiency of the array structure and the digital filter.

Multiplier involves three stages of processing of inputs, namely, (a) partial product generation, (b) partial reduction, and (c) final addition with carry propagation. Typical implementation of the multiplier utilizes the carry save array multiplication which requires more computation leading to delayed output and consumes more power. To improve the performance of the multiplier, compressors are utilized which performs reduction of partial products in parallel. This helps in increasing the performance by reducing the interconnect delays and the glitches associated with logic transitions; which leads to reduced power consumption.

As an example for *N*-bit multiplier, the *N* numbers of rows of partial products are divided into *N*/2 number of rows and computed in parallel. For *N* = 8, compressors are used in three stages. Compressors are the complex standard library cells which have 5 inputs and 3 outputs. Among the 5 inputs, one of the inputs is the intermediate carry-in from previous stage and, of the 3 outputs, one is the current column sum; and the other 2 are intermediate carry (also called horizontal carry since it propagates horizontally through the compressors in a row) and vertical carry (saved for next stage computation).

There are several compressor architectures discussed in the past [6, 7]. Compressors can be implemented using two full adders, basic tree cells, and with existing standard compressor cell of the library.

**Figure 2:**
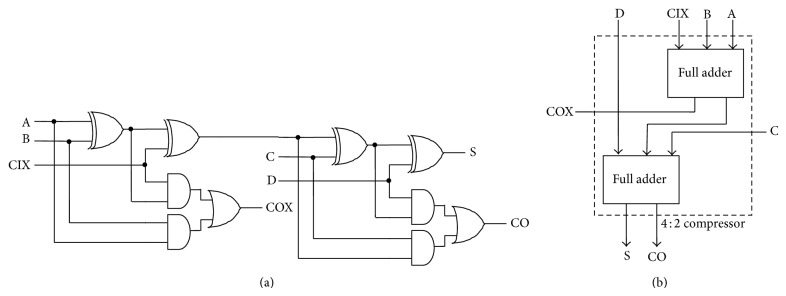
(a) Neil and Harris compressor architecture [7]. (b) Compressor architecture using full adder [6].

Figures 2(a) and 2(b) show the gate level architecture of the compressor architectures. As mentioned in the Introduction, the design oriented architectures provide higher efficiency, and similarly the conventional compressor architecture limits its usage in low power constrained designs.

In the architecture of [7], compressor cell contains more interconnects, which forms the basis for the interconnect delays and increased glitches. On the other side the architecture may behave faster but it is unused for the low power applications since trade-off of delay is also accepted. Moreover to have the reduced leakage power the cells should have higher number of transistors in the stack, and here in this architecture it is only two (for XOR and OR gates). The regular compressor architecture also consists of inverters (e.g., embedded in AND & OR logics) in the critical path, which leads to logic transitions and increases the power consumption. Thus the regular compressor architecture of Figure 2(a) is clearly unsuited for low power applications.

Figure 2(b) shows the compressor architecture built with the full adder. This compressor architecture might have fewer interconnects but the sum and carry paths are shared and it requires larger drive strength to drive the signal; this results in the high power consumption. Such cells are suited for the timing constraints as the higher drive strengths will boost the timing performance of the cell.

**Figure 3:**
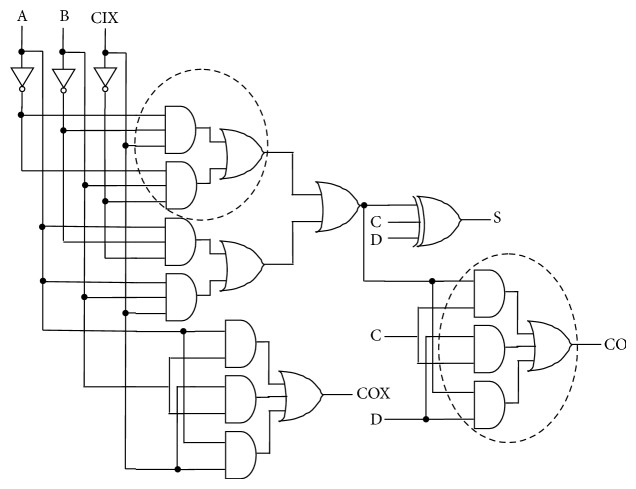
Proposed compressor architecture.

Figure 3 shows the gate level architecture of the proposed compressor architecture which consists of complex cells. The AND-OR logic based complex cells with three levels of transistor stacks are utilized. Higher transistor stacks increase the ON resistance between the supply rails and help in reducing the leakage power. Since complex cells are used; the number of gates required for the compressors is also less, which minimizes the interconnect delays and the associated glitches. The increase in transistor stacks increases the delay and is recovered to certain extent by reducing the shared logic between the sum and carry paths. That is, intermediate carry is generated in parallel. The proposed logic has minimum number of inverters in the critical path.

Thus the use of compressor architectures in the multiplier behaves similar to the characteristics of the compressor architectures as they are more in numbers and forms the basis for the different design constraints for the conventional and proposed compressor architectures. Thus the multiplier with proposed compressor architecture can be utilized for low leakage power constraints. And such multipliers are utilized in the systolic array of the digital filter for biomedical application (QRS detector of ECG analysis) where leakage power is the critical factor, since most parts of the monitoring systems will be in standby mode for larger time.

Outcomes of proposed architecture are

- design oriented/specific architectures,
- low power compressor architecture having
  1. minimum interconnect delays and associated glitches,
  2. minimum number of inverters in critical path,
  3. parallel horizontal carry generation,
  4. reduced leakage power,
  5. concept holding good and true for any digital logic system since datapath architectural innovations,
- low power multiplier for systolic array based digital filter,
- impact of datapath architectural optimizations at the subsystem level.

## 3. Results and Discussions

The impact of power aware and datapath architecture is addressed briefly in this paper. To carry out the analysis, gate level architectures were described using verilog HDL and verified the functionality through waveform editor of Mentor Graphics ModelSim simulator. With suitable design constraints the design was synthesized in Synopsys Design compiler by mapping to TSMC's 65 nm technological library node. Standard ASIC design methodology was followed to benchmark the results [8–10].

Table 1 shows the results of the existing and proposed compressor architecture. As mentioned in Section 2, the results in Table 1 prove that the design specific architectures are more efficient than the generic architectures; that is, the proposed compressor architecture leaks less power than the existing compressor architecture. Minimum interconnect delay and associated glitches have reduced the delay and dynamic power consumption. As the number of compressors in the design increases the efficiency of the proposed compressor architecture also increases.

Table 2 gives the results of the 8-bit multiplier having existing and proposed compressor architectures. The concepts similar to the compressor architecture were applied to the adders associated within the multipliers while implementing the multiplier architecture with proposed compressor architecture to compensate for the increased area in compressor architecture.

Table 2 suggests that the impact of proposed compressor architecture holds true at the multiplier level. This proves that the datapath architectural optimizations, as per the constraints, are efficient and impact is also higher. The more the number of compressors, the higher the impact.

Similarly the multiplier having existing and proposed compressor architectures and proposed full adder in proposed multiplier are integrated into the multipliers of the systolic array of digital filter. The results at the digital filter level are obtained by applying similar input constraints to existing and proposed filters; and they are tabulated in Table 3.

Thus it can be concluded that the architectures designed as per the design constraints are efficient and the impact of the datapath optimizations is also higher. As mentioned in the Introduction the trade-offs are accepted when certain constraints are achieved for particular applications.

**Figure 4:**
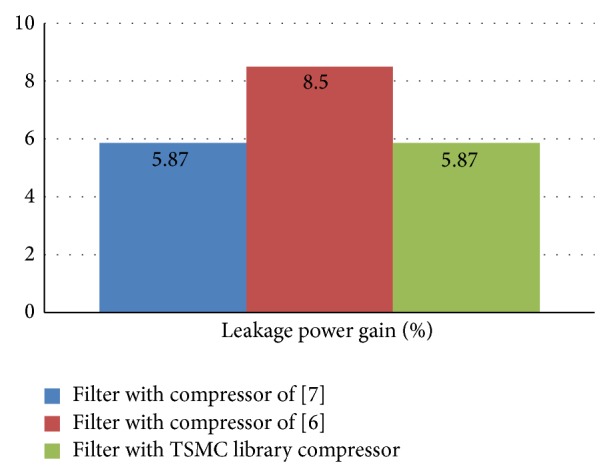
Comparison of leakage power % gain of proposed filter and existing filter.

Figure 4 gives the percentage gain of the leakage power of the proposed filter against the existing architectures. As the paper describes the low leakage power constraints, hence results of the leakage power are reproduced as in chart in Figure 4. It can be observed that the proposed compressor architecture has impacted the filter level and enables using it as the base architecture for low leakage power constraint designs or applications. This suggests that the proposed concept holds good and true at all hierarchical levels of the system design.

From Tables 1–3, it can be observed that the leakage power has been reduced at all hierarchical levels. This suggests that the proposed architecture behaves similarly at all hierarchical levels and since it is an architectural innovation, the proposed architecture will behave similarly for any bit widths.

## 4. Conclusion

Low power DSP systems result in newer DSP applications like palm held devices, portable digital assistants, and also mainly in sensor and monitoring systems in biomedical field. Proposed design illustrates the impact of the power aware architectures on the power constrained systems. A digital filter used for ECG analysis is implemented and proved that the datapath architectural optimizations as per the design constraints are more efficient than the generic architectures. The proposed filter design has reduced the significant amount of leakage power than all other filter architectures.

It is observed during this work that the communication between the DSP Algorithm designers and low power VLSI designers is important to achieve satisfactory results required by the end consumer.

## Figures and Tables

**Table 1:** Existing and proposed compressor architecture results.

Design	Compressor			
	Existing [7]	Existing [6]	Existing (TSMC library compressor cell)	Proposed
Area	27.36	20.16	16.55	28.08
Delay	0.41	0.21	0.29	0.34
DP	6.12	5.125	4.37	4.977
LP	0.284	0.307	0.24	0.236
TP	6.404	5.432	4.61	5.213

Note: “area” in square microns; “delay” in nanoseconds; “DP” dynamic power in microwatt; “LP” leakage power in microwatt; “TP” total power in microwatt.

**Table 2:** Results of multiplier with existing and proposed compressor architecture.

Design	Multiplier			
	With existing compressor of [7]	With existing compressor of [6]	Existing (TSMC library compressor cell)	Proposed
Area	859.68	711.36	668.759	801
Delay	2.48	1.72	1.47	2.72
DP	177.8	173.3	162.8	171.9
LP	8.45	9.28	8.45	6.88
TP	186.25	182.58	171.25	178.78

Note: “area” in square microns; “delay” in nanoseconds; “DP” dynamic power in microwatt; “LP” leakage power in microwatt; “TP” total power in microwatt.

**Table 3:** Results of digital filter with existing and proposed compressor architecture.

Design	Digital filter			
	With existing compressor of [7]	With existing compressor of [6]	Existing (TSMC library compressor cell)	Proposed
Area	9793.8	9200.52	9027.72	9559
Delay	3.45	3.45	3.45	3.63
DP	434.7	432.8	429.2	432.9
LP	104	107	104	97.9
TP	538.7	539.8	533.2	530.8

Note: “area” in square microns; “delay” in nanoseconds; “DP” dynamic power in microwatt; “LP” leakage power in microwatt; “TP” total power in microwatt.
